# Evaluating the use of large language model in identifying top research questions in gastroenterology

**DOI:** 10.1038/s41598-023-31412-2

**Published:** 2023-03-13

**Authors:** Adi Lahat, Eyal Shachar, Benjamin Avidan, Zina Shatz, Benjamin S. Glicksberg, Eyal Klang

**Affiliations:** 1 Department of Gastroenterology, Chaim Sheba Medical Center, Affiliated to Tel Aviv University, Tel Aviv, Israel; 2 Hasso Plattner Institute for Digital Health, Icahn School of Medicine at Mount Sinai, New York, NY USA; 3 The Sami Sagol AI Hub, ARC Innovation Center, Chaim Sheba Medical Center, Affiliated to Tel-Aviv University, Tel Aviv, Israel

**Keywords:** Computational biology and bioinformatics, Gastroenterology, Medical research

## Abstract

The field of gastroenterology (GI) is constantly evolving. It is essential to pinpoint the most pressing and important research questions. To evaluate the potential of chatGPT for identifying research priorities in GI and provide a starting point for further investigation. We queried chatGPT on four key topics in GI: inflammatory bowel disease, microbiome, Artificial Intelligence in GI, and advanced endoscopy in GI. A panel of experienced gastroenterologists separately reviewed and rated the generated research questions on a scale of 1–5, with 5 being the most important and relevant to current research in GI. chatGPT generated relevant and clear research questions. Yet, the questions were not considered original by the panel of gastroenterologists. On average, the questions were rated 3.6 ± 1.4, with inter-rater reliability ranging from 0.80 to 0.98 (*p* < 0.001). The mean grades for relevance, clarity, specificity, and originality were 4.9 ± 0.1, 4.6 ± 0.4, 3.1 ± 0.2, 1.5 ± 0.4, respectively. Our study suggests that Large Language Models (LLMs) may be a useful tool for identifying research priorities in the field of GI, but more work is needed to improve the novelty of the generated research questions.

## Introduction

The field of gastroenterology (GI) is constantly evolving, with new advances in technology and research offering insights into the diagnosis and treatment of GI conditions^1^. In order to continue pushing the field forward, it is essential to identify the most important research questions that require further investigation.

Traditionally, the identification of research priorities in GI has relied on expert opinion and consensus-building among researchers and clinicians. However, this approach may not always capture the full range of potential research questions.

In recent years, the use of natural language processing (NLP) techniques has gained popularity as a means of identifying research priorities. In particular, large language models (LLMs), such as chatGPT, that are trained on vast amounts of text data have shown promise in suggesting research questions based on their ability to understand human-like language^2,3^.

Previous publications evaluating large language models in various other fields of research included for example the evaluation of the commonsense ability of GPT, BERT, XLNet, and RoBERTa^4^ with promising results, evaluation of CODEX, GPT-3 and GPT-J for code generation capabilities^5^,evaluation of three approaches to personalizing a language model^6^, and evaluating the text to Structured Query Language (SQL) capabilities of CODEX^7^.

In this paper, we evaluate the use of newly-released chatGPT in identifying top research questions in the field of GI. We focus on four key areas: inflammatory bowel disease (IBD), the microbiome, AI in GI, and advanced endoscopy in GI. We prompted the model to generate a list of research questions for each topic. These questions were then reviewed and rated by experienced gastroenterologists to assess their relevance and importance.

We aimed to evaluate the potential of chatGPT as a tool for identifying important research questions in the field of GI. By utilizing the latest advances in NLP, we hope to shed light on the most pressing and important research questions in the field, and to contribute to the continued advancement of GI research.

## Methods

The study was conducted by utilizing chatGPT, version released on December 15, a recently introduced LLM (Nov 2022). The model was trained by OpenAI ^2^, chatGPT was queried on four key topics in GI: inflammatory bowel disease (IBD), microbiome, AI in GI, and advanced endoscopy in GI and was requested to identify the most relevant research questions in each topic.

A total of 5 research questions were generated for each topic, resulting in a total of 20 research questions. These questions were then reviewed and rated separately by a panel of experienced gastroenterologists with expertise in the respective topic areas. The panel consisted of 3 gastroenterologists, two of them with over 20 years of experience, and one with over 30 years of experience. All gastroenterologists work in an academic tertiary medical center and are the authors of dozens of academic research publications in Gastroenterology, and together cover most sub-specialized in Gastroenterology: IBD experts, motility, nutrition, and advanced endoscopy. Research key topics were selected in a consensus between all Gastroenterologists and two AI experts.

ChatGPT was prompted with four key topics related to the field of gastrointestinal research. For each topic, a new thread was started in order to eliminate any potential bias from previous conversations and to ensure that the generated responses were directly related to the current prompt. The four topics were framed as research questions, and were carefully crafted to elicit relevant information about the most important questions in the four chosen topics of gastrointestinal research. Supplementary Table 1 presents the prompts used to generate the research questions in each topic.

The gastroenterologists were asked to rate each research question on a scale of 1–5, with 5 being the most important and relevant to current research in the field of GI. The mean rating ± SD for each research question was calculated. Each question was graded according to 4 parameters: relevance originality, clarity and specificity.

To determine inter-rater reliability, we used the intraclass correlation coefficient (ICC)^8^ (see statistical analysis).

All data were collected and analyzed using standard statistical methods. The research questions generated by chatGPT were compared to the current research questions being addressed in the field of GI, as identified through a comprehensive review of the literature. This allowed for an assessment of the novelty and relevance of the questions generated by chatGPT.

### Statistical analysis

In this study, the mean, standard deviation, and median were utilized to describe the data. The Intra-Class analysis (Two-Mixed model, Absolute Agreement) or the Intraclass Correlation Coefficient (inter-rater agreement) were employed to assess the data. To determine the significance of the difference in grades among the four research topics (each with 20 questions), a Wilcoxon test for non-parametric paired samples was conducted. All calculations were performed using IBM SPSS Statistical Package version 28.

To assess the reliability of the rating process, the ICC was calculated. The ICC was selected as the type of reliability estimate, with the ratings made by each of the three observers being treated as separate items. The ICC value was interpreted as follows: a value of 0 indicated no agreement among the ratings, a value of 1 indicated perfect agreement, and values between 0 and 1 indicated some degree of agreement, with higher values indicating greater agreement. Additionally, the mean ratings and standard deviations of the three observers were compared using the SPSS Explore function, and the correlations among the ratings made by the three observers were examined.

## Results

A diverse range of research questions was generated by the chat GPT. A panel of 3 expert gastroenterologists evaluated the created questions. All questions suggested by the chatGPT on the topics of IBD, microbiome, AI, and advanced endoscopy and their ratings by the expert gastroenterologists are shown in Supplementary Table 2.

In order to establish the validity of the expert ratings in this study, we first assessed the inter-rater agreement among the evaluators. To eliminate the potential confounding influence of intraclass variability, we employed a Two-Mixed Model with random people effects and fixed measures effects to compute the Correlation Coefficient (ICC) among the raters. The ICC values obtained in this analysis ranged from 0.8 to 0.98 and were statistically significant (*p* < 0.001), indicating a high level of reliability in the expert ratings. This strong agreement among the raters suggests that their assessments can be considered reliable indicators of expert opinion.

Agreement among the experts according to topics is shown in Table 1.

**Table 1:** Correlation coefficient among experts.

Subject	Intraclass correlation	95% Confidence interval		Sig
		Lower bound	Upper bound	
Advanced endoscopy	0.928	0.837	0.970	< .001
AI	0.949	0.893	0.978	< .001
IBD	0.979	0.953	0.992	< .001
Microbiome	0.981	0.959	0.992	< .001
Overall	0.961	0.941	0.975	< .001

The results of the expert evaluation showed that chatGPT was able to generate research questions that were most relevant to the field of IBD, with the majority of questions receiving a relevance rating of 5—the highest rate, and a mean grade of 4.9 ± 0.26. In terms of clarity, chatGPT performed very well, with most questions receiving a rating of 4 or 5, and a mean grade of 4.8 ± 0.41. For specificity, the chatGPT reached a mean grade of 2.86 ± 0.64—a moderately good result. However, for originality, all grades were very low—with a mean of 1.07 ± 0.26.

When assessing microbiome-related topics, results were similar to those achieved for IBD.

As in IBD, grades reached almost the maximum for relevance and clarity, and the minimum for originality. Question 1 was even identical for both topics.

The mean ± SD for relevance originality, clarity, and specificity were: 4.93 ± 0.26, 1.13 ± 0.35, 4.93 ± 0.26, and 3.13 ± 0.64, respectively.

Results for AI and advanced endoscopy show the same trend- high relevance and clarity, good specificity but the lowest originality. Mean results for AI concerning all the above measures are 5 ± 0, 4.33 ± 0.89, 3.2 ± 0.67 and 1.87 ± 0.99, respectively.

The mean results for advanced endoscopy for relevance, clarity, specificity, and originality were: 4. ± 0.89, 4.47 ± 0.74, 3.2 ± 0.77 and 1.73 ± 1.03, respectively.

As shown in Table 2, the same trend was continuous in the mean and median grades across all topics, with high grades for relevance and clarity, good for specificity and very low for originality.

**Table 2:** Mean and median grades for all topics across all parameters.

	Clarity	Originality	Relevance	Specificity
N	20	20	20	20
Mean	4.6	1.5	4.9	3.1
Median	4.7	1.2	5.0	3.3
SD	0.51	0.72	0.38	0.48

**Figure 1:**
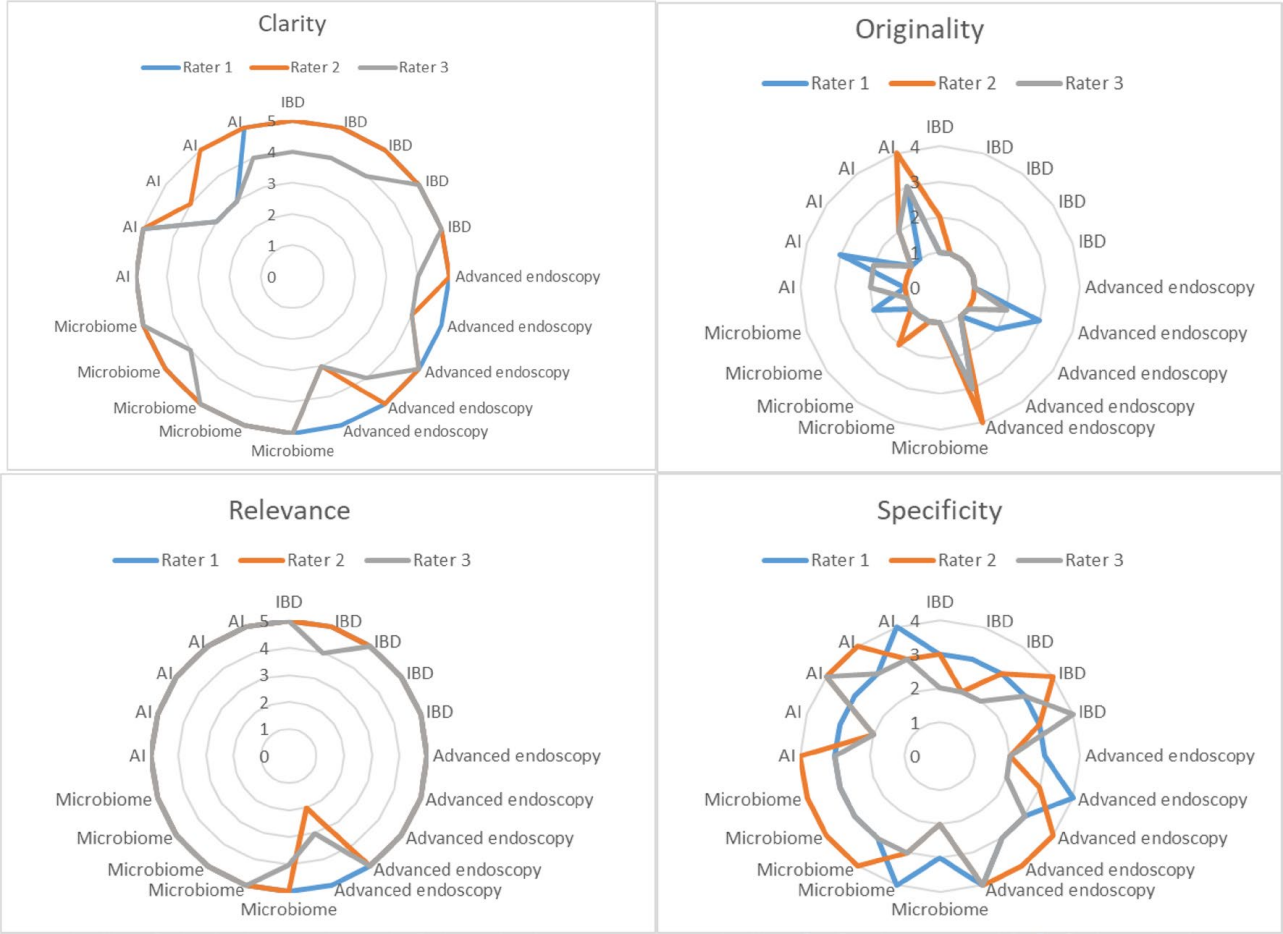
Level of inter-rater agreement and the mean grades in all categories for every topic. The figure illustrates the level of inter-rater agreement and the mean grades in all categories for every topic. When the curves representing the ratings of different evaluators are closer together within the circle, it indicates a higher level of agreement among them. The further the curve is from the outer edge of the diagram, the higher the grades given by the evaluators. The monotonic nature of the curves suggests that the raters are consistent between their assessments.

Figure 1 illustrates the level of inter-rater agreement and the mean grades in all categories for every topic. When the curves representing the ratings of different evaluators are closer together within the circle, it indicates a higher level of agreement among them. The further the curve is from the outer edge of the diagram, the higher the grades given by the evaluators. The monotonic nature of the curves suggests that the raters are consistent between their assessments.

**Figure 2:**
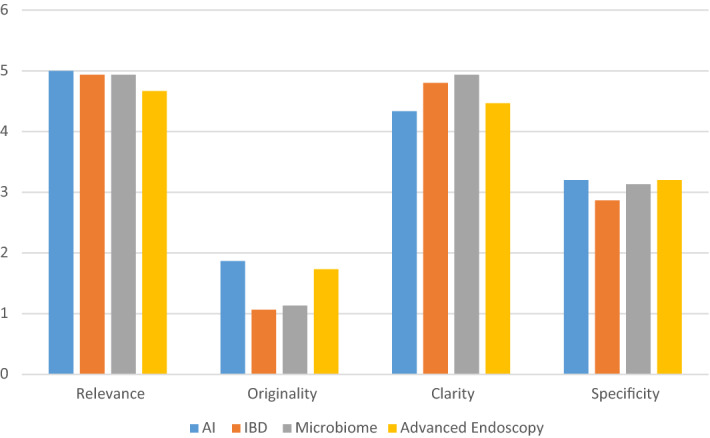
Mean scores for each research topic and category, as rated by all readers.

In general, ChatGPT demonstrated excellent results in terms of Clarity and Relevance, satisfactory performance in terms of Specificity, but inadequate performance in terms of Originality. Figure 2 presents the mean scores for all readers for each category and each research topic.

## Discussion

When evaluating chatGPT for generating important research questions in IBD, microbiome, AI in gastroenterology, and advanced endoscopy in gastroenterology, we found that the model has the potential to be a valuable tool for generating high-quality research questions in these topics. In all the examined topics, chatGPT was able to produce a range of relevant, clear, and specific research questions, as evaluated by a panel of expert gastroenterologists. However, none of the questions was original. In fact, in terms of originality, the chatGPT showed poor performance.

Overall, the results of our evaluation show that chatGPT has the potential to be a valuable resource for researchers. Its ability to generate a diverse range of high-quality research questions can help to advance the field by providing researchers with novel ideas for investigation. However, further research and development are needed to enhance chatGPT's ability in terms of originality.

The results of the work reflect the general ability of ChatGPT to produce any type of text. Similar properties of clarity and relevance were part of the reward model of ChatGPT's original training, in which humans rated several outputs of the model according to their preferences. Thus, the model is able to produce outputs that are also rated as clear and relevant by other human raters. The limitation of the originality of the results is mentioned first on ChatGPT´s homepage^2^, and is further emphasized in our current study.

One potential area for future research is to explore the use of chatGPT in conjunction with other natural language processing techniques, such as topic modeling^9^, to identify relevant research areas and generate more focused and specific research questions. Additionally, further studies could investigate the use of chatGPT in other subfields of gastroenterology, such as hepatology and gastrointestinal surgery, to assess its potential. Furthermore, we believe chatGPT can be relevant to many other fields of medical research.Importantly, the originality of the research topic received very low scores. This result highlights a key disadvantage of the large language models: NLP models are trained on a vast amount of text data and are able to generate responses based on these data^10–12^. While they are able to provide accurate and informative responses to a wide range of questions, these responses are not original or unique in the sense that they are not generated from their own experiences or insights. Instead, they are based on preexisting information and language patterns that the NLP models have learned from the data they were trained on. As a result, the responses generated by a language model are often not regarded as original ideas or insights.

In this study we measured the intraclass correlation coefficient (ICC) between 3 experienced gastroenterologists, in order to evaluate the consistency and reliability of the questions’ assessments. A high ICC indicates that the observations made by different observers are highly consistent, which suggests that the results of the study are accurate.

Despite the promising results of this study, there are limitations that should be considered when interpreting the findings. First, the expert panel that generated research questions consisted of only three gastroenterologists and two AI experts, and the panel that evaluated the questions consisted of three gastroenterologists. Though highly experienced, the results may not be representative of the broader community of researchers in these fields. Nevertheless, the results are solidified by the high degree of inter-observer agreement, which underscores the validity of the conclusions reached.

Further studies with larger and more diverse panels of experts would be needed to confirm the generalizability of these results.

Second, the evaluation of chatGPT's performance was based on subjective ratings by the expert panel, which may be subject to bias and variability. Objective measures, such as the citation frequency or impact factor of current academic papers focusing on the same topics of the research questions generated by chatGPT, would provide a more robust assessment of its performance.

However, research questions often involve complex issues that cannot be easily quantified, such as the relevance of a question or the originality of a question in the existing literature.

Therefore, subjective judgment is an essential component of the evaluation of research questions and helps to ensure that the questions are relevant, clear, feasible, original, evidence-based, and valid, taking into account the complex and context-specific nature of research questions.

Furthermore, the quality of a research question can also be influenced by human values, such as ethical considerations, societal impact, and personal beliefs. These values cannot be easily quantified, and are best evaluated through subjective judgment.

Third, this study focused on the performance of chatGPT in generating research questions in specific subfields of gastroenterology, but did not investigate its potential for generating research questions in other areas of medicine or science. Further research is needed to evaluate chatGPT's performance in a wider range of domains.

Fourth, we used a single set of prompts for each of the four research topics to generate the research questions. Given that ChatGPT is sensitive to tweaks in the input, more experiments with different prompts would have been valuable in order to fully evaluate the potential of ChatGPT to generate diverse research questions. Additionally, we only used one instance of ChatGPT, and it is possible that the results could have been different with another instance of the model or a different language model. Further research is needed to determine the generalizability of our results to other models and contexts.

It is noteworthy that the text summarization capabilities of GPT-3 were recently evaluated and displayed impressive results utilizing traditional benchmarks^13^. Currently, as the utilization of the chatbot GPT is rapidly increasing, a vast amount of data is accumulating at a rapid pace regarding its various capabilities^14,15^.

In conclusion, our evaluation of chatGPT as a research idea creator for four key topics in gastroenterology—inflammatory bowel disease, microbiome, AI in gastroenterology, and advanced endoscopy—showed promising results. ChatGPT was able to generate high-quality research questions in these fields, demonstrating its potential as a valuable tool for advancing the field of gastroenterology. While further research and development is needed to enhance chatGPT’s performance in terms of relevance and originality, its ability to generate a diverse range of clear and specific research questions has the potential to significantly contribute to the advancement of gastroenterology. Overall, chatGPT has the potential to be a valuable tool for researchers in the field of gastroenterology specifically and in other medical fields in general, and we believe it is worth further exploration and development.

## Supplementary Information

Supplementary Information.

## Data Availability

The authors declare that there is no relevant data available for this study. All data used in the analysis and preparation of this manuscript have been included in the manuscript.
