# Supplementary material for: Evaluating the use of large language model in identifying top research questions in gastroenterology
Source: Sci Rep. 2023 Mar 13;13:4164. doi: 10.1038/s41598-023-31412-2 (PMC10011374; doi:10.1038/s41598-023-31412-2)
Supplement: Supplementary file 1 — Supplementary Information. [file 41598_2023_31412_MOESM1_ESM.docx]

**Supplementary files**

**Supplementary Table 1-** Prompts for ChatGPT-Generated Research Questions on the Four Key Topics in the Field of Gastrointestinal Research.

| **Topic** | **Prompt** |
| --- | --- |
| IBD | Please write in bullets five titles for important unsolved research questions in the field of inflammatory bowel disease. The titles should fit papers in top-tier gastrointestinal journals. |
| Microbiome | Please write in bullets five titles for important unsolved research questions in the field of microbiome. The titles should fit papers in top-tier gastrointestinal journals. |
| AI | Please write in bullets five titles for important unsolved research questions in the field of AI in gastroenterology. The titles should fit papers in top-tier gastrointestinal journals. |
| Advanced endoscopy | Please write in bullets five titles for important unsolved research questions in the field of advanced endoscopy in gastroenterology. The titles should fit papers in top-tier gastrointestinal journals. |

**Supplementary Table 2- Questions generated by ChatGPT in 4 major topics and their grades**

| **subject** | **Question** | **parameter** | **Observer 1** | **Observer 2** | **Observer 3** | **MEAN (between observers, OR intervariability)** |
| --- | --- | --- | --- | --- | --- | --- |
| IBD | The Efficacy and Safety of New Therapeutic Approaches for IBD | Relevance | 5 | 5 | 5 | 5.0 |
| IBD | The Efficacy and Safety of New Therapeutic Approaches for IBD | Originality | 1 | 1 | 1 | 1.0 |
| IBD | The Efficacy and Safety of New Therapeutic Approaches for IBD | Clarity | 5 | 5 | 4 | 4.7 |
| IBD | The Efficacy and Safety of New Therapeutic Approaches for IBD | Specificity | 3 | 3 | 2 | 2.7 |
| **IBD** | **The Efficacy and Safety of New Therapeutic Approaches for IBD** | **MEAN (within observer, or intravariability)** | **3.5** | **3.5** | **3** |  |
|  |  |  |  |  |  |  |
| IBD | The Genetic Basis of IBD: Identifying Novel Susceptibility Loci | Relevance | 5 | 5 | 4 | 4.7 |
| IBD | The Genetic Basis of IBD: Identifying Novel Susceptibility Loci | Originality | 1 | 1 | 1 | 1.0 |
| IBD | The Genetic Basis of IBD: Identifying Novel Susceptibility Loci | Clarity | 5 | 5 | 4 | 4.7 |
| IBD | The Genetic Basis of IBD: Identifying Novel Susceptibility Loci | Specificity | 3 | 2 | 2 | 2.3 |
| **IBD** | **The Genetic Basis of IBD: Identifying Novel Susceptibility Loci** | **MEAN (within observer, or intravariability)** | **3.5** | **3.25** | **2.75** |  |
|  |  |  |  |  |  |  |
| IBD | The Role of Environmental Factors in IBD: A Systematic Review | Relevance | 5 | 5 | 5 | 5.0 |
| IBD | The Role of Environmental Factors in IBD: A Systematic Review | Originality | 1 | 1 | 1 | 1.0 |
| IBD | The Role of Environmental Factors in IBD: A Systematic Review | Clarity | 5 | 5 | 5 | 5.0 |
| IBD | The Role of Environmental Factors in IBD: A Systematic Review | Specificity | 3 | 4 | 3 | 3.3 |
| **IBD** | **The Role of Environmental Factors in IBD: A Systematic Review** | **MEAN (within observer, or intravariability)** | **3.5** | **3.75** | **3.5** |  |
|  |  |  |  |  |  |  |
| IBD | The Role of Microbiota in the Development and Progression of IBD | Relevance | 5 | 5 | 5 | 5.0 |
| IBD | The Role of Microbiota in the Development and Progression of IBD | Originality | 1 | 2 | 1 | 1.3 |
| IBD | The Role of Microbiota in the Development and Progression of IBD | Clarity | 5 | 5 | 4 | 4.7 |
| IBD | The Role of Microbiota in the Development and Progression of IBD | Specificity | 3 | 3 | 2 | 2.7 |
| **IBD** | **The Role of Microbiota in the Development and Progression of IBD** | **MEAN (within observer, or intravariability)** | **3.5** | **3.75** | **3** |  |
|  |  |  |  |  |  |  |
| IBD | The Role of the Immune System in the Pathogenesis of IBD: A Comprehensive Review | Relevance | 5 | 5 | 5 | 5.0 |
| IBD | The Role of the Immune System in the Pathogenesis of IBD: A Comprehensive Review | Originality | 1 | 1 | 1 | 1.0 |
| IBD | The Role of the Immune System in the Pathogenesis of IBD: A Comprehensive Review | Clarity | 5 | 5 | 5 | 5.0 |
| IBD | The Role of the Immune System in the Pathogenesis of IBD: A Comprehensive Review | Specificity | 3 | 3 | 4 | 3.3 |
| **IBD** | **The Role of the Immune System in the Pathogenesis of IBD: A Comprehensive Review** | **MEAN (within observer, or intravariability)** | **3.5** | **3.5** | **3.75** |  |
|  |  |  |  |  |  |  |
| Advanced endoscopy | Developing personalized treatment plans using advanced endoscopy in gastroenterology | Relevance | 5 | 5 | 5 | 5.0 |
| Advanced endoscopy | Developing personalized treatment plans using advanced endoscopy in gastroenterology | Originality | 2 | 1 | 1 | 1.3 |
| Advanced endoscopy | Developing personalized treatment plans using advanced endoscopy in gastroenterology | Clarity | 5 | 5 | 5 | 5.0 |
| Advanced endoscopy | Developing personalized treatment plans using advanced endoscopy in gastroenterology | Specificity | 3 | 4 | 3 | 3.3 |
| **Advanced endoscopy** | **Developing personalized treatment plans using advanced endoscopy in gastroenterology** | **MEAN (within observer, or intravariability)** | **3.75** | **3.75** | **3.5** |  |
|  |  |  |  |  |  |  |
| Advanced endoscopy | Evaluating the ethical implications of using advanced endoscopy in gastrointestinal care | Relevance | 5 | 2 | 3 | 3.3 |
| Advanced endoscopy | Evaluating the ethical implications of using advanced endoscopy in gastrointestinal care | Originality | 3 | 4 | 3 | 3.3 |
| Advanced endoscopy | Evaluating the ethical implications of using advanced endoscopy in gastrointestinal care | Clarity | 5 | 3 | 3 | 3.7 |
| Advanced endoscopy | Evaluating the ethical implications of using advanced endoscopy in gastrointestinal care | Specificity | 4 | 4 | 4 | 4.0 |
| **Advanced endoscopy** | **Evaluating the ethical implications of using advanced endoscopy in gastrointestinal care** | **MEAN (within observer, or intravariability)** | **4.25** | **3.25** | **3.25** |  |
|  |  |  |  |  |  |  |
| Advanced endoscopy | Exploring the potential of advanced endoscopy in predicting and preventing gastrointestinal diseases | Relevance | 5 | 5 | 5 | 5.0 |
| Advanced endoscopy | Exploring the potential of advanced endoscopy in predicting and preventing gastrointestinal diseases | Originality | 3 | 1 | 2 | 2.0 |
| Advanced endoscopy | Exploring the potential of advanced endoscopy in predicting and preventing gastrointestinal diseases | Clarity | 5 | 4 | 4 | 4.3 |
| Advanced endoscopy | Exploring the potential of advanced endoscopy in predicting and preventing gastrointestinal diseases | Specificity | 4 | 3 | 2 | 3.0 |
| **Advanced endoscopy** | **Exploring the potential of advanced endoscopy in predicting and preventing gastrointestinal diseases** | **MEAN (within observer, or intravariability)** | **4.25** | **3.25** | **3.25** |  |
|  |  |  |  |  |  |  |
| Advanced endoscopy | Improving diagnostic accuracy of advanced endoscopy techniques in gastroenterological imaging | Relevance | 5 | 5 | 5 | 5.0 |
| Advanced endoscopy | Improving diagnostic accuracy of advanced endoscopy techniques in gastroenterological imaging | Originality | 1 | 1 | 1 | 1.0 |
| Advanced endoscopy | Improving diagnostic accuracy of advanced endoscopy techniques in gastroenterological imaging | Clarity | 5 | 5 | 4 | 4.7 |
| Advanced endoscopy | Improving diagnostic accuracy of advanced endoscopy techniques in gastroenterological imaging | Specificity | 3 | 2 | 2 | 2.3 |
| **Advanced endoscopy** | **Improving diagnostic accuracy of advanced endoscopy techniques in gastroenterological imaging** | **MEAN (within observer, or intravariability)** | **3.5** | **3.25** | **3** |  |
|  |  |  |  |  |  |  |
| Advanced endoscopy | Investigating the effectiveness of advanced endoscopy-assisted interventions in gastrointestinal surgeries | Relevance | 5 | 5 | 5 | 5.0 |
| Advanced endoscopy | Investigating the effectiveness of advanced endoscopy-assisted interventions in gastrointestinal surgeries | Originality (1-5) | 1 | 1 | 1 | 1.0 |
| Advanced endoscopy | Investigating the effectiveness of advanced endoscopy-assisted interventions in gastrointestinal surgeries | Clarity (1-5) | 5 | 5 | 4 | 4.7 |
| Advanced endoscopy | Investigating the effectiveness of advanced endoscopy-assisted interventions in gastrointestinal surgeries | Specificity (1-5) | 3 | 4 | 3 | 3.3 |
| **Advanced endoscopy** | **Investigating the effectiveness of advanced endoscopy-assisted interventions in gastrointestinal surgeries** | **MEAN (within observer, or intravariability)** | **3.5** | **3.75** | **3.25** |  |
|  |  |  |  |  |  |  |
| AI | Developing personalized treatment plans using AI in gastroenterology | Relevance | 5 | 5 | 5 | 5.0 |
| AI | Developing personalized treatment plans using AI in gastroenterology | Originality | 1 | 1 | 1 | 1.0 |
| AI | Developing personalized treatment plans using AI in gastroenterology | Clarity | 3 | 4 | 3 | 3.3 |
| AI | Developing personalized treatment plans using AI in gastroenterology | Specificity | 3 | 4 | 4 | 3.7 |
| **AI** | **Developing personalized treatment plans using AI in gastroenterology** | **MEAN (within observer, or intravariability)** | **3** | **3.5** | **3.25** |  |
|  |  |  |  |  |  |  |
| AI | Evaluating the ethical implications of using AI in gastrointestinal care | Relevance | 5 | 5 | 5 | 5.0 |
| AI | Evaluating the ethical implications of using AI in gastrointestinal care | Originality | 3 | 4 | 3 | 3.3 |
| AI | Evaluating the ethical implications of using AI in gastrointestinal care | Clarity | 5 | 5 | 4 | 4.7 |
| AI | Evaluating the ethical implications of using AI in gastrointestinal care | Specificity | 4 | 3 | 3 | 3.3 |
| **AI** | **Evaluating the ethical implications of using AI in gastrointestinal care** | **MEAN (within observer, or intravariability)** | **4.25** | **4.25** | **3.75** |  |
|  |  |  |  |  |  |  |
| AI | Exploring the potential of AI in predicting and preventing gastrointestinal diseases | Relevance | 5 | 5 | 5 | 5.0 |
| AI | Exploring the potential of AI in predicting and preventing gastrointestinal diseases | Originality | 3 | 1 | 2 | 2.0 |
| AI | Exploring the potential of AI in predicting and preventing gastrointestinal diseases | Clarity | 5 | 5 | 5 | 5.0 |
| AI | Exploring the potential of AI in predicting and preventing gastrointestinal diseases | Specificity | 3 | 2 | 2 | 2.3 |
| **AI** | **Exploring the potential of AI in predicting and preventing gastrointestinal diseases** | **MEAN (within observer, or intravariability)** | **4** | **3.25** | **3.5** |  |
|  |  |  |  |  |  |  |
| AI | Improving diagnostic accuracy of AI algorithms in gastroenterological imaging | Relevance | 5 | 5 | 5 | 5.0 |
| AI | Improving diagnostic accuracy of AI algorithms in gastroenterological imaging | Originality | 1 | 1 | 2 | 1.3 |
| AI | Improving diagnostic accuracy of AI algorithms in gastroenterological imaging | Clarity | 5 | 5 | 5 | 5.0 |
| AI | Improving diagnostic accuracy of AI algorithms in gastroenterological imaging | Specificity | 3 | 4 | 3 | 3.3 |
| **AI** | **Improving diagnostic accuracy of AI algorithms in gastroenterological imaging** | **MEAN (within observer, or intravariability)** | **3.5** | **3.75** | **3.75** |  |
|  |  |  |  |  |  |  |
| AI | Investigating the effectiveness of AI-assisted interventions in gastrointestinal surgeries | Relevance | 5 | 5 | 5 | 5.0 |
| AI | Investigating the effectiveness of AI-assisted interventions in gastrointestinal surgeries | Originality | 1 | 2 | 2 | 1.7 |
| AI | Investigating the effectiveness of AI-assisted interventions in gastrointestinal surgeries | Clarity | 3 | 5 | 3 | 3.7 |
| AI | Investigating the effectiveness of AI-assisted interventions in gastrointestinal surgeries | Specificity | 3 | 4 | 3 | 3.3 |
| **AI** | **Investigating the effectiveness of AI-assisted interventions in gastrointestinal surgeries** | **MEAN (within observer, or intravariability)** | **3** | **4** | **3.25** |  |
|  |  |  |  |  |  |  |
| microbiome | Identifying the factors that influence the composition of the gut microbiome in different individuals | Relevance | 5 | 5 | 5 | 5.0 |
| microbiome | Identifying the factors that influence the composition of the gut microbiome in different individuals | Originality | 1 | 1 | 1 | 1.0 |
| microbiome | Identifying the factors that influence the composition of the gut microbiome in different individuals | Clarity | 5 | 5 | 5 | 5.0 |
| microbiome | Identifying the factors that influence the composition of the gut microbiome in different individuals | Specificity | 4 | 3 | 3 | 3.3 |
| **microbiome** | **Identifying the factors that influence the composition of the gut microbiome in different individuals** | **MEAN (within observer, or intravariability)** | **3.75** | **3.5** | **3.5** |  |
|  |  |  |  |  |  |  |
| microbiome | The mechanisms by which the gut microbiome contributes to the development of obesity and metabolic syndrome | Relevance | 5 | 5 | 5 | 5.0 |
| microbiome | The mechanisms by which the gut microbiome contributes to the development of obesity and metabolic syndrome | Originality | 1 | 2 | 1 | 1.3 |
| microbiome | The mechanisms by which the gut microbiome contributes to the development of obesity and metabolic syndrome | Clarity | 5 | 5 | 5 | 5.0 |
| microbiome | The mechanisms by which the gut microbiome contributes to the development of obesity and metabolic syndrome | Specificity | 3 | 4 | 3 | 3.3 |
| **microbiome** | **The mechanisms by which the gut microbiome contributes to the development of obesity and metabolic syndrome** | **MEAN (within observer, or intravariability)** | **3.5** | **4** | **3.5** |  |
|  |  |  |  |  |  |  |
| microbiome | The potential of probiotics and prebiotics as therapeutic interventions for gut microbiome-related disorders | Relevance | 5 | 5 | 5 | 5.0 |
| microbiome | The potential of probiotics and prebiotics as therapeutic interventions for gut microbiome-related disorders | Originality | 1 | 1 | 1 | 1.0 |
| microbiome | The potential of probiotics and prebiotics as therapeutic interventions for gut microbiome-related disorders | Clarity | 5 | 5 | 4 | 4.7 |
| microbiome | The potential of probiotics and prebiotics as therapeutic interventions for gut microbiome-related disorders | Specificity | 3 | 4 | 3 | 3.3 |
| **microbiome** | **The Genetic Basis of IBD: Identifying Novel Susceptibility Loci** | **MEAN (within observer, or intravariability)** | **3.5** | **3.75** | **3.25** |  |
|  |  |  |  |  |  |  |
| microbiome | The role of the gut microbiome in the development and progression of colorectal cancer | Relevance | 5 | 5 | 5 | 5.0 |
| microbiome | The role of the gut microbiome in the development and progression of colorectal cancer | Originality | 2 | 1 | 1 | 1.3 |
| microbiome | The role of the gut microbiome in the development and progression of colorectal cancer | Clarity | 5 | 5 | 5 | 5.0 |
| microbiome | The role of the gut microbiome in the development and progression of colorectal cancer | Specificity | 3 | 4 | 3 | 3.3 |
| **microbiome** | **The role of the gut microbiome in the development and progression of colorectal cancer** | **MEAN (within observer, or intravariability)** | **3.75** | **3.75** | **3.5** |  |
|  |  |  |  |  |  |  |
| microbiome | The role of the gut microbiome in the development of inflammatory bowel disease | Relevance | 5 | 5 | 4 | 4.7 |
| microbiome | The role of the gut microbiome in the development of inflammatory bowel disease | Originality | 1 | 1 | 1 | 1.0 |
| microbiome | The role of the gut microbiome in the development of inflammatory bowel disease | Clarity | 5 | 5 | 5 | 5.0 |
| microbiome | The role of the gut microbiome in the development of inflammatory bowel disease | Specificity | 3 | 2 | 2 | 2.3 |
| **microbiome** | **The role of the gut microbiome in the development of inflammatory bowel disease** | **MEAN (within observer, or intravariability)** | **3.5** | **3.25** | **3** |  |
